# Reduction in Hospital Admissions Associated with Coronary Events during the COVID-19 Pandemic in the Brazilian Private Health System: Data from the UNIMED-BH System

**DOI:** 10.1590/0037-8682-0174-2021

**Published:** 2021-07-02

**Authors:** Bruno Ramos Nascimento, Luisa Campos Caldeira Brant, Ana Cristina Teixeira Castro, Luiz Eduardo Vieira Froes, Antonio Luiz Pinho Ribeiro, Renato Azeredo Teixeira, Larissa Vilela Cruz, Cynthia Bicalho Maluf Araújo, Charles Ferreira Souza, Eduardo Tomaz Froes, Soraya Diniz Souza

**Affiliations:** 1 Universidade Federal de Minas Gerais, Hospital das Clínicas, Serviço de Cardiologia e Cirurgia Cardiovascular e Centro de Telessaúde, Belo Horizonte, MG, Brasil.; 2 Universidade Federal de Minas Gerais, Faculdade de Medicina, Belo Horizonte, MG, Brasil.; 3 UNIMED-BH Cooperativa Medica, Serviço de Atendimento Móvel, Belo Horizonte, MG, Brasil.; 4 Universidade José do Rosário Vellano, Faculdade de Medicina, Belo Horizonte, MG, Brasil.; 5 UNIMED-BH Cooperativa Médica, Centro Médico, CPS Barreiro e Central de Consultas On-line, Belo Horizonte, MG, Brasil.; 6 UNIMED-BH Cooperativa Médica, Gerência de Desenvolvimento de Informações para o Negócio, Belo Horizonte, MG, Brasil.

**Keywords:** Cardiovascular disease, Coronary artery disease, Covid-19, Admissions, Mortality

## Abstract

**INTRODUCTION:**

We aimed to evaluate the impact of the new coronavirus disease 2019 on coronary hospitalizations in the Brazilian private health system.

**METHODS:**

Data on coronary admissions in 2020 and a 2-year historical series were collected from the UNIMED-BH insurance system.

**RESULTS:**

Admission rates in 2020 reduced by 26% (95%CI, 22-30) in comparison with 2018/2019, markedly from March to May (37%) compared to the peak of the pandemic (June-September, 19%). Mortality was higher in 2020 (5.4%, 95%CI 4.5-6.4) than in 2018/2019 (3.6%, 95%CI 3.2-4.1).

**CONCLUSIONS:**

There was a significant decrease in coronary admissions, with higher mortality during the COVID-19 pandemic.

Since the beginning of the new coronavirus disease 2019 (COVID-19) pandemic in March 2020, strict social containment measures have been adopted worldwide, affecting the structure of provision of medical services, especially for non-emergent conditions, and emergency systems^1^ ^-^ ^3^. In several locations, a marked decrease in hospital admissions associated with acute coronary syndromes (ACS) was reported during the pandemic. In Italy, a significant decrease in hospital admissions due to ACS was observed^4^, in parallel with a 58% increase in out-of-hospital cardiac arrest, which was strongly associated with COVID-19 incidence^5^. Similarly, an average of 38% reduction in US cardiac catheterization laboratory activations due to ST-elevation myocardial infarction was observed early in the pandemic^6^, similar to the 40% reduction reported in Spain^7^ and 48% decrease in the weekly hospitalization rates for ACS in California (USA)^8^. Therefore, the outcomes associated with coronary events may also have been impacted.

In metropolitan Belo Horizonte, Southeast Brazil, social isolation policies were issued on March 18, 2020. UNIMED-BH is a local, non-profit private medical system, cooperatively run by physicians, currently with 5,700 doctors and a network of 355 accredited services, covering approximately 1.31 million clients (22% of the population). Being one of the largest medical cooperatives in Brazil with a robust electronic database, the analysis of its records may help depict the effects of the pandemic on cardiovascular admissions in Brazilian private health facilities. We aimed to evaluate the impact of COVID-19 on hospitalization and death rates due to coronary events in the Brazilian large-scale UNIMED-BH private health system.

 We conducted an observational study with retrospective administrative data collection. Data were exported from the UNIMED-BH central database, hosted in the Management of Business Information Development (*Gerência de Desenvolvimento de Informações para o Negócio,* GDIN), and entries were identified. All UNIMED-BH owned and accredited hospitals and clinics are connected to the administrative database, which is also linked to quality of care, audit and reimbursement. We collected demographic and clinical data from patients admitted in 2018, 2019, and 2020 for urgent and elective coronary events (acute coronary syndromes, coronary interventions, clinical management of coronary artery disease, and invasive diagnostic procedures for coronary artery disease), according to the *Terminologia Unificada da Saúde Suplementar (TUSS)* (Supplementary Health Unified Terminology) procedures (codes 30911079, 30912105, 30912180, 30912040, 30912032, 30912113, and 30912261) with final diagnoses by the Diagnosis Related Groups (DRG) method^9^ (keywords: *ACUTE MYOCARDIAL INFARCTION", "ANGINA PECTORIS, “"CORONARY INTERVENTION, ““CARDIAC CATHETERIZATION, ““CORONARY SYNDROME, “"CORONARY ATEROSCLEROTIC DISEASE*” and related diagnoses) (**Supplement 1:** http://data.nber.org/drg/drgdesc08.pdf), crosslinked to ICD-10 codes. The study endpoints (hospital admissions, all-cause death, and length of hospital stay) as well as COVID-19 laboratory tests (real-time polymerase chain reaction and serology) were retrospectively recorded from the patients’ registries. As humans were not directly involved in the study, UNIMED-BH provided the Data Usage Agreement, and the research protocol was approved by the Institutional Review Board of Universidade Federal de Minas Gerais (no. 31095320.2.0000.5149).

Data were exported from the GDIN system as an Excel (Microsoft Corporation, Redmond, VA, US) file and imported to SPSS® software (version 23.0) for Mac OSX (SPSS Inc., Chicago, IL, USA) for statistical analysis. As an exploratory study, no pre-specified sample size calculation was performed, and we considered the total number of patients admitted with coronary events from March 18 (epidemiological week 12) to September 30 (epidemiological week 39), 2020, and endpoints were collected up to 15 days after the last inclusion (October 15, 2020). Categorical variables, expressed as numbers and percentages, were compared between groups (historical series 2018/2019 vs. 2020) using the chi-square test, whereas continuous data, expressed as mean ± SD or median or Q1/Q3 (25%/75%), were compared using Student’s unpaired t-test or the Mann-Whitney U test, as appropriate. Changes in rates of hospital admissions per 100,000 clients were measured by comparing the 2020-point estimate and the expected point-estimate measured by applying the mean rates of 2018/2019, considering the mean covered population in each time interval. A two-tailed significance level of 0.05 was used in the study.

During the 196-day period, the number of coronary admissions in 2018, 2019, and 2020 was 2,789, 3,519, and 2,348, respectively, and patients had a median age of 67 (58-76) years, with 59% being men. A total of 12.2% of admissions occurred in UNIMED-BH-owned hospitals. The characteristics of the admissions in each year are detailed in Table 1.

**TABLE 1:** Characteristics of hospital admissions associated with coronary events in the UNIMED-BH system, from March to September, 2018 to 2020.

Variable:	2018	2019	2020
Total clients (N, million)	1.259	1.268	1.278
Sex (male, %)	57.6	59.0	59.3
Age (years, median [IQR])	67 [58 - 75]	67 [58 - 76]	67 [57 - 75]
Coronary admissions (total) (N, rate/100,000)*	2,789 (222/100,000)	3,519 (278/100,000)	2,348 (184/100,000)
Repeated admissions (N, % [95% CI])	632 (22.7 [21.1 - 24.2])	1086 (30.9 [29.3 - 32.4])	619 (26.4 [24.6 - 28.2])
Coronary admissions (March - May 2020) (N)	1,159	1,329	794
Coronary admissions (June - Sept 2020) (N)	1,617	2,172	1,538
Primary PCI (N, % [95% CI])	138 (4.9 [4.2 - 5.8])	144 (4.1 [3.5 - 4.8])	134 (5.7 [4.8 - 6.7])
Activation of ambulance system (N, % [95% CI])	452 (16.2 [14.9 - 17.6])	577 (16.4 [15.2 - 17.7])	522 (22.2 [20.6 - 24.0])
UNIMED-BH owned hospitals (N, % [95% CI])	278 (10.0 [8.9 - 11.1])	405 (11.5 [10.5 - 12.6])	372 (15.8 [14.4 - 17.4])
Length of hospital stay (days, mean ± SD)	6.8 ± 10.1	6.6 ± 8.9	6.9 ± 8.9
Death (N, % [95% CI])	87 (3.1 [2.5 - 3.8])	141 (4.0 [3.4 - 4.7])	126 (5.4 [4.5 - 6.4])

**Abbreviations: CI:** confidence interval; **IQR:** interquartile range; **N/A:** not applicable; **PCI:** percutaneous coronary intervention; **SD:** standard deviation. *Mean UNIMED-BH clients (million): **2018:** 1.259, **2019:** 1.268, **2020:** 1.278.

The mean length of hospital stay was 6.7±9.3 days, and 27% had more than one admission in 3 years, without significant differences in readmission rates from 2018/2019 to 2020. In 2020, more admissions followed activations of the UNIMED-BH emergency ambulance system (22.2% vs. 16.3%), and the proportion of primary percutaneous coronary interventions (PCIs) was also higher (5.7% vs. 4.5%) (Table 1).

The adjusted rates of admissions were 221, 278, and 184/100,000 clients, respectively, resulting in a significant reduction of 26% (95% CI 22-30) in 2020 compared to the historical series. The reduction was more pronounced from March to May (37%, 95% CI 31-42), immediately following social isolation policies, compared to the peak of the pandemic (June-September, 19%; 95% CI 14-25) (Figure 1). Only 53 patients admitted in 2020 were diagnosed with COVID-19, and nine of them died.

**FIGURE 1:**
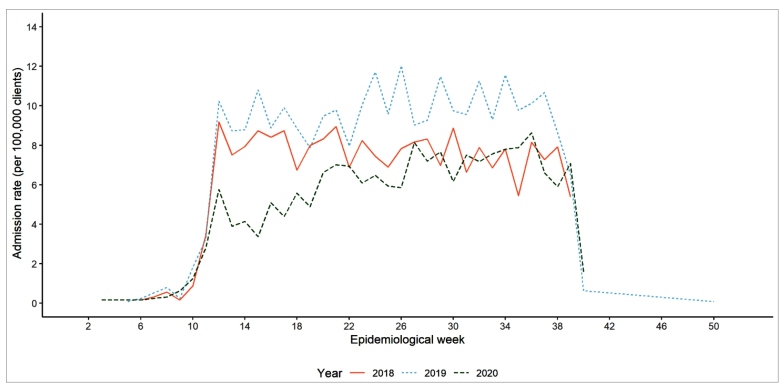
Admission rates for coronary events by epidemiological week, in 2018, 2019, and 2020, considering the mean covered population (clients) in each month. From the UNIMED-BH administrative database.

 In-hospital mortality was also significantly higher in 2020 (5.4%, 95% CI 4.5-6.4) than in 2018/2019 (3.6%, 95% CI 3.2-4.1), despite the similar age of patients (67 [57-76] vs. 67 [58-76]) and length of hospital stay (6.9±8.9 vs. 6.7±9.5 days). Mortality was higher in 2020, even after exclusion of COVID-19 deaths (5.1% vs. 3.6%). In 2020, mortality was similar between March to May (5.5%, 95% CI 4.1-7.4) and June to September (5.1%, 95% CI 4.0-6.3), despite a longer hospital stay in the first period (7.8±9.1 vs. 5.9±6.6 days).

Our data, from one of the largest private health insurance systems in Brazil, suggest that the COVID-19 pandemic had a remarkable impact on hospital admissions for coronary events in Brazil, especially in the beginning, following the issue of social isolation policies, suggesting that individuals may not have sought hospital care due to regulatory measures or due to fear of contracting COVID-19 at hospitals. Compared to the 2-year series (2018/2019), mortality rates were higher, which may suggest an impact of the pandemic on clinical outcomes for acute diseases.

The reduction in hospitalizations related to coronary artery disease has been reported in many countries, such as Italy and the US^4^ ^-^ ^6^ however, in the present study, this reduction was not proportional to primary PCI and ambulance activation. This information, together with an increase in mortality rates during the period, raises the hypothesis that hospitalized patients may have had more severe presentations of ACS and possibly may have been later presenters, resulting in prolonged door-to-guidewire times and delayed implementation of guideline-based interventions, leading to more in-hospital deaths and complications^10^. 

Considering the interaction of COVID-19 with the cardiovascular system, promoting inflammation and increasing thrombogenicity^11^, it may seem counterintuitive to find a reduction in ACS in the pandemic context. Actually, a previous study in six Brazilian capital cities showed that although deaths due to ACS reduced during the pandemic, excess mortality due to cardiovascular diseases (CVDs) increased in the out-of-hospital setting and due to non-specific cardiovascular conditions, suggesting that ACS could have been underdiagnosed during the period^12^. Being a private system, UNIMED-BH had an anticipated response to the pandemic through a multimodal telemedicine system, expansion of COVID-19 beds in owned and accredited hospitals, and continued educational interventions^13^. Despite this, the reorganization of emergency and intensive care sectors, deferral of elective procedures and appointments, and avoidance of medical care affected the pattern of CVD admissions. Although the system did not collapse, the occupancy of hospital beds was high during the first peak of the pandemic. 

Importantly, the effect of the pandemic on coronary disease may just be starting^14^. The interruption of CVD primary and secondary prevention pathways, and treatment of chronic disease, along with a rise in traditional risk factors, such as smoking and physical inactivity^15^, may result in an increase in cardiovascular events in the months and years to come. Public campaigns to inform the population about the importance of seeking medical assistance in the presence of signs and symptoms of acute CVD and where to go, in case of reorganization of health systems, are fundamental to mitigate further impact of the pandemic on cardiovascular health. Moreover, novel strategies to reassume CVD prevention and the promotion of healthy lifestyles must be prioritized in public health policies.

Our study has several limitations. First, it reports the findings of a single system, limiting the generalization of these findings. Second, DRG was the only coding system applied, and only the final main diagnoses were considered, limiting insights into the underlying health conditions. Moreover, administrative data were not detailed enough for the application of severity scores for ACS, which could allow for the comparison of disease presentation in the pre-and post-pandemic periods. Finally, as the civil registry was not accessed, the underlying causes of death were not available, nor data about patients who died of presumed CVD prior to hospital admission. Despite these limitations, this report using data from one of the largest private health systems in Latin America (1.31 million clients) provides reliable information about the magnitude of the impact of COVID-19 on hospital management of CVD and points towards a possible impact on health outcomes.

In conclusion, there was a significant decrease in admissions due to coronary events during the COVID-19 pandemic in a large Brazilian metropolitan area, in parallel with increased in-hospital mortality. Deferral of elective procedures, reorganization of health systems and emergency services, and avoidance of medical care presumably resulted in delayed presentation and unfavorable outcomes. 
